# Editorial Notes and Miscellany

**Published:** 1912-05

**Authors:** 


### Editorial Notes and Miscellany.

MARRIED at Marlin, Texas, April 24th (ult.), Evelyn, the accomplished daughter of Dr. and Mrs. S. P. Rice, to Albert M. Hutchinson, all of Marlin.

SURGICAL HOSPITAL WANTED.—Surgeon desires to negotiate for practice and hospital in Texas city or town. Address, E. B. Y., 507 West Thirty-third Street, Austin, Texas.

Johnny (excitedly)—"Mommer! there's a man in the hall kissing the cook!"

Mamma—"Oh, horrors!"

Johnny (gleefully)—"April fool, Mommer! It's only papa!"

DOCTOR, in case you sent your bill to a patron repeatedly, for several years' service, and he paid no attention to it,—ignored it; what would you do? Say, a small amount (like a subscription to the "Red Back," for instance), do you think he would be treating you just right?

WE REGRET TO ANNOUNCE the death, in San Antonio, March 8th, of Ben Manes, aged 16, eldest son of Dr. O. B. Manes, late of that city. We extend to the doctor our sincere condolence on the loss of his son, a most promising young man. Dr. Manes has just removed to Coleman, Texas, his old home, where the remains of his son were interred, and will practice medicine there.

INTANGIBLE ASSETS.—Miss Spry—"Senator, what do you lawyers mean by 'intangible assets?'"

Senator J. Bird—"The word is derived from the Latin, 'tangere'—to touch. Tangible assets are one's possessions that can be touched, seen, handled. Thus, if you have anything that can not be touched, seen ——"

Miss Spry (hurriedly)—"Oh, I see! What time is it, Senator?"

THE COMMUNICATION elsewhere published in this issue of the "Red Back," "Pulmonary Consumption Cured by Persistent Walking, and Without Drugs," is written by one of the best known and most popular physicians of Texas, long since retired from practice. He is remarkable—amongst other excellent qualities—for his modesty, and gives me the paper on condition that it be published anonymously. He is a man of large experience, being now near three score and ten, and a close observer, and his statements are entitled to respect and consideration. Read the article.

---

"NAUGHTY" 606.—Why do so many doctors say "six—aught—six," when they mean, "six—naught—six? and "consider?" when they mean "deem?"

---

WITHIN A DAY OR TWO after this issue is in the hands of members, the big State Medical Association convention will be held at Waco, 7th, 8th and 9th (inst.). A large attendance is expected, and arranged for. Reduced rates as usual. For full particulars, see the April number of the *State Association Journal.*

---

TO THE COURT OF CRIMINAL APPEALS (with apology to Bret Harte).—

Now, I hold it is not proper for a member of the court
To call his brother judge an ass, or words of like import;
Nor should the learned member who happens to be meant
Reply by hurling epithets to any great extent.

---

GREATER NEW YORK NUMBER.—In June the *American Journal of Surgery* will issue a number composed of original contributions from men of recognized prominence in the medical profession residing in Greater New York. Among those to contribute are: Herman J. Boldt, C. N. Dowd, Meddaugh Dunning, Wm. S. Gottheil, E. L. Keys, Jr., Howard Lilienthal, Chas. H. May, Willy Meyer, Robt. T. Morris, S. Lewis Pilcher, John O. Polak, James P. Tuttle, James P. Warbasse, and others.

Contributions from these well known men should make this issue of particular interest and value.

---

GOOD.—A woman to be chief of the new Child's Bureau. The President has appointed Julia C. Lathrop, of Chicago, an assistant to Jane Addams of Hull House, to the position of Chief of the new Children's Bureau, in the Department of Commerce and

Labor. She is the first woman ever appointed a bureau chief under the government.

---

| | |
|---|---|
| TEXAS MEDICAL JOURNAL, one year | $1 00 |
| *American Journal Clinical Medicine* | 2 00 |
| "Strange Case of Dr. Bruno" | 1 50 |
| | $4 50 |

All for $3.00. Renewals count if arrears are paid.

---

WAS SURE HE KNEW IT.—The physiology class in a country school was studying about the backbone.

Teacher—"What are the pieces of cartilage for between the vertebræ?"

A little boy raised his hand.

"Well, Eddie, you may tell us," the teacher said.

"To take the jars off the jumps," answered the triumphant Eddie.—From *National Monthly*.

